# Does Fluid Temperature Affect Corneal Endothelium-Descemet Membrane Scroll Formation? An In Vitro Study

**DOI:** 10.4274/tjo.72368

**Published:** 2018-10-31

**Authors:** Yusuf Koçluk, Emine Alyamaç Sukgen, Selim Cevher

**Affiliations:** 1 Adana Numune Training and Research Hospital, Ophthalmology Clinic, Adana, Turkey; 2 Ereğli State Hospital, Ophthalmology Clinic, Konya, Turkey

**Keywords:** Descemet membrane endothelial keratoplasty, Descemet membrane, balanced salt solution, Descemet membrane unfolding time, donor cornea

## Abstract

**Objectives:**

To investigate whether unfolding time of Descemet membrane (DM) graft rolls changes at various fluid temperatures.

**Materials and Methods:**

The study was prospective, *ex vivo*, and experimental. The study was conducted at the tertiary center for corneal disease in Adana Numune Training and Research Hospital between June 2014 and June 2015. DMs were divided into 4 categories according to baseline roll tightness and these were distributed among 4 different groups using 4 different balanced salt solution (BSS) temperatures (8, 16, 23, and 36 °C). Sixteen donor corneas were obtained from the hospital eye bank.

**Results:**

DM roll formations may vary according to the donor cornea received. Some form tighter rolls while others can form a more open roll. No differences in roll tightness were observed in any of the DM rolls after 5 or 10 minutes in the different BBS temperatures. In all groups, neither tightening nor opening was observed in DM roll formations.

**Conclusion:**

Different BSS temperatures were found to have no effects on DM unfolding time in this study.

## Introduction

Descemet membrane endothelial keratoplasty (DMEK) was first described by Melles et al.^1,2^ as the replacement of diseased endothelium and Descemet membrane (DM) using an isolated endothelium DM layer without adherent corneal stroma. Although the Descemet stripping automated endothelial keratoplasty (DSAEK) procedure involves well standardized and reproducible graft preparation and unfolding, DMEK remains challenging. The main step of the procedure, which involves unfolding the lamella to attach the graft to the posterior stroma, can be especially difficult. This step involves most of the manipulations to the graft.^3^

DMEK provides faster and more complete visual rehabilitation compared to DSAEK.^4,5,6^ However, graft preparation and unfolding are less standardized. In DMEK, the graft preparation phase, injection of DM into the anterior chamber, and all the unfolding phases in the anterior chamber are performed in a fluid medium. We hypothesized that graft unfolding could be affected by the temperature of the fluid medium.

We observed a spontaneous opening of tight DM rolls that were difficult to open in the DMEK surgeries of some patients, and thought that it may be due to the warming effect of the microscope light. If DMEK surgery is prolonged, the microscope light can have a thermal effect. The major protein in DM is type IV collagen. With heat, the structure of collagen is disrupted by strong oscillations that break the bonds between molecules, and this may affect DM unfolding time.^7^ Although there was no animal model or other research showing that temperature change can affect DM roll tightness, we investigated the effect of temperatures up to normal body temperatures on DM rolls in a laboratory setting.

This experimental study investigated whether the unfolding time of cornea DMEK graft rolls which could not be used due to positive serology changed in various fluid temperatures.

## Materials and Methods

This study was prospective, *ex vivo*, and experimental in nature. Ethical approval was obtained from Adana Numune Training and Research Hospital, where the study was conducted. The study was carried out with 16 donor corneas which were obtained from the eye bank of the tertiary center for corneal disease in Adana Numune Training and Research Hospital between June 2014 and June 2015. The donors’ ages were noted. The donor corneas were not appropriate for implantation due to positive results in serological tests (HbsAg^+^ or antiHCV^+^).

Corneoscleral buttons were excised and stored in a corneal chamber which contained Eusol-C (AlchimiA, Viale Austria, Italy) at 4 °C for 14 days. DM tissues were found to have no endothelial pathology by biomicroscopic examination (there was no evidence of corneal endothelial dystrophy or folds, and no history of intraocular surgery). The death-to-preservation time was not longer than 12 hours without refrigeration and the tissue was used within 7 days of harvesting.

### Donor Preparation

**Figure 1:**
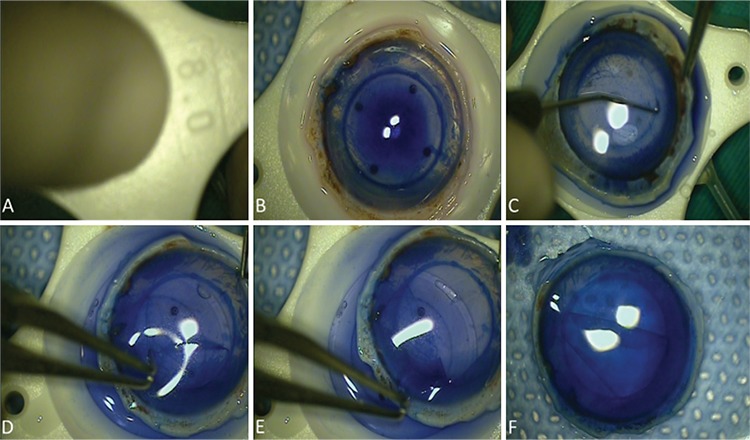
Harvesting of Descemet membrane (DM) roll. (A) Superficial trephination on the endothelial side; (B) staining DM edges with trypan blue; (C) finding edge of DM using Sinskey hook; (D) stripping toward the center using tying forceps; (E) completion of DM stripping; (F) restaining DM roll with trypan blue DM: Descemet membrane

All tissues were prepared by a single surgeon (Y.K.) in the operating room using the submerged cornea technique immediately before evaluation as described previously.^8^ Corneoscleral buttons were positioned onto a Barron vacuum punch to prevent shifting during DM preparation (Katena Products, Inc., New Jersey, USA). All DM grafts underwent superficial trephination using an 8.0 mm punch, which is one of the most preferred diameters in DMEK surgery. The DM edges were stained with 0.06% trypan blue solution and the donor rim was filled with 23 °C balanced salt solution (BSS). Stripping was performed in this medium using tying forceps, working from edge to center, and the DM was harvested. The DM roll was then restained with trypan blue for 60 s (Figure 1).

### DM Rolls

The DM grafts were divided into 4 categories according to initial roll tightness. Accordingly, 1/4 DM roll was very tight (about 1-2 mm width), 2/4 DM roll had about 3-4 mm width, 3/4 DM roll had about 5-6 mm width, and 4/4 DM roll was nearly completely open (about 7-8 mm width).

Using tying forceps, the prepared DM rolls were placed into closed glass containers containing BSS at different temperatures. Changes in DM roll formations and sizes were observed and photographed by ophthalmic surgical microscope at the beginning, at 5 minutes, and at 10 minutes.

The temperature of the operating room and liquids (relatively) in the room may vary between 22 and 24 °C during eye surgery.^9^ The ambient temperature was ~23°C in the operating room where the current study was performed. In another study, it was determined that the mean temperature in the anterior chamber was 23.6 °C.^10^ Therefore, 23 °C is a logical BSS temperature for stripping of DM graft. Considering the temperature range between 8 °C (4-8 °C is storage temperature of donor tissue) and 36 °C (human body temperature) suitable for this study, we evaluated temperatures of 8, 16, 23, and 36 °C to determine the optimal BSS temperature for DM grafts.

The study was conducted in 4 different groups, using 4 different BSS temperatures: 3 (20.0%) DM grafts were placed in 8 °C BSS (Group 1), 4 (26.7%) in 16 °C BSS (Group 2), 4 (26.7%) in 23 °C BSS (Group 3), and 4 (26.7%) in 36 °C BSS (Group 4). BSS temperatures in all groups were checked with a thermometer and kept stable throughout the 10-minute observation period. Changes in fold tightness of the DM grafts in BSS at different temperatures were observed for 10 minutes. In DMEK surgery, shorter DM graft unfolding time is desirable to ensure less endothelial loss and favorable postoperative prognosis. Thus, the effects of BSS temperature on the unfolding time of DM grafts in 10 minutes were observed and recorded in this study.

### Statistical Analysis

SPSS version 16.0 software (SPSS, Inc., Chicago, IL) was utilized in the analysis of the data. The analysis included nonparametric tests. Median and range or mean ± standard deviations (SD) were demonstrated through descriptive statistics. The distribution of proportions was analysed using chi-square distribution. Statistical significance was accepted as p<0.05.

## Results

The median donor age (16 corneas of 8 donors) was 69 years (range, 65-71 years). The male/female ratio was 6 (37.5%)/10 (62.5%). There were no significant differences between the groups in terms of age and gender (p=0.114, p=0.362, respectively).

The median time from death to corneal removal was 7 hours (range, 5-9 hours), while the median time from preservation to surgery was 5.5 days (range, 5-7 days). There were no statistical differences between the groups in terms of time from death to corneal removal or preservation to surgery (p=0.687, p=0.887, respectively).

Cause of death was cerebrovascular disease in 3 donors, myocardial infarction in 2 donors, and chronic kidney disease in 3 donors. It was thought that cause of death would affect DM roll formation. However, there were no statistical differences between the groups in terms of the donors’ cause of death (p=0.238).

All tissues were stripped in the operating room and prepared immediately before evaluation. The median DM peeling time was 7.0 minutes (range, 5-20 minutes), with no statistically significant differences between the groups (p=0.946). Of the 16 corneas, the DM roll could not be obtained from only 1 (in Group 1). This lacerated DM was excluded from the study.

**Figure 2:**
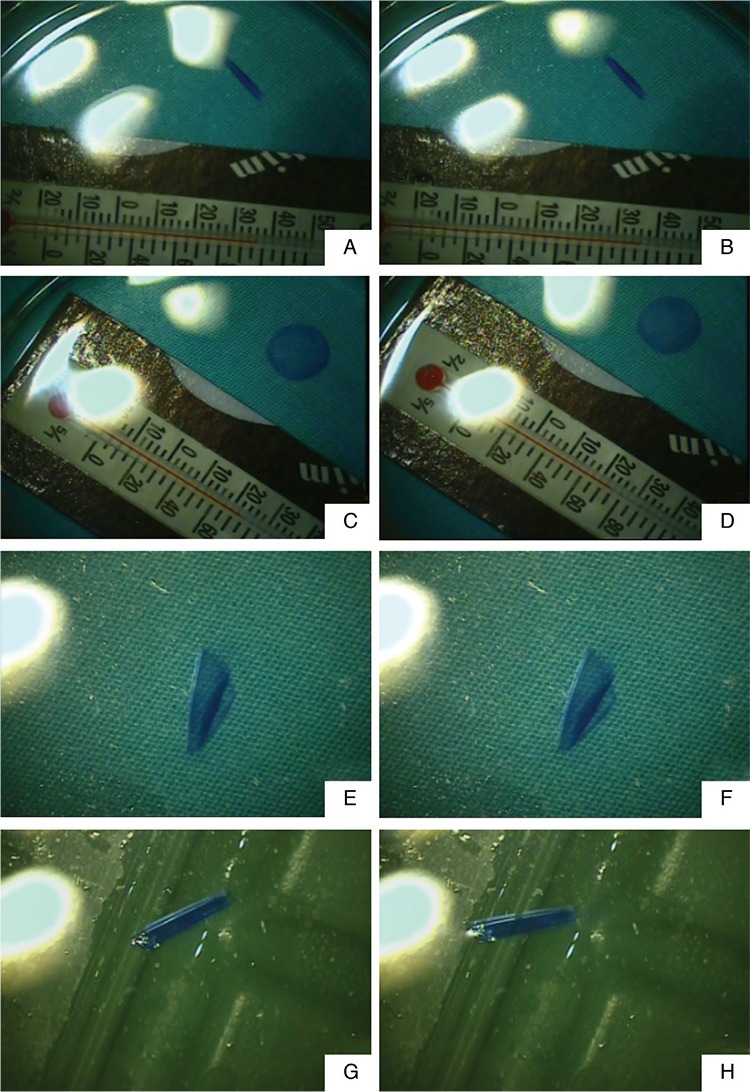
Demonstration of DM roll unfolding at baseline and the 10th minute after putting in different BSS temperatures; (A) DM roll in the 36 °C BSS temperature at baseline, (B) DM roll at the 10th minute after putting into the 36 °C BSS temperature, (C) DM roll in the 23 °C BSS temperature at baseline, (D) DM roll at the 10th minute after putting into the 23 °C BSS temperature, (E) DM roll in the 16 °C BSS temperature at baseline, (F) DM roll at the 10th minute after putting into the 16 °C BSS temperature, (G) DM roll in the 8 °C BSS temperature at baseline, (H) DM roll at the 10th minute after putting into the 8 °C BSS temperature. DM: Descemet membrane, BSS: Balanced salt solution

Prepared DM roll formations can vary according to the donor cornea. In the current study, some formed a tighter roll, while others formed a looser roll. At time of collection, there was no statistical difference between the groups in DM roll tightness in the 23 °C BSS (p=0.273) (Table 1). In all groups, no differences were observed in the DM roll formations and width of the tissues in the different temperatures of BSS after 5 and 10 minutes. The DM graft rolls prepared in the 23 °C BSS temperature were not affected by increasing or decreasing the BSS temperature for 10 minutes. In all groups, neither tightening nor opening was observed in DM roll formations. Figure 2 displays the images of some DM rolls in groups at 0 and 10 minutes.

## Discussion

DMEK preparation might be affected by some systemic conditions. Research shows that DMEK preparation can have higher failure rates with tissues from donors with diabetes mellitus (especially with longer duration of the disease) and hyperlipidemia or obesity. It is reported that failure rate can be reduced by eliminating the tissues from donors either with diabetes mellitus or with hyperlipidemia or obesity.^11^ The related literature indicates general rate of failure as 5.2%.^8,12^ In our study, we could not obtain DM graft from only one donor cornea (6.25%). There were lacerations resulting from the tightly cohesive nature of the stromal surface. This donor’s cause of death was chronic kidney disease with diabetes mellitus, which is consistent with the information in the literature.^11^

Donor age, systemic diseases, and cause of death may have an impact on DM graft preparation, complications during harvesting, and DM roll formation.^3,11,13^ In the current study, preoperative findings were similar and differences between groups were not statistically significant. Therefore, we were able to investigate only the effects of different BSS temperatures on DM roll formation in this study.

A statistically significant correlation was reported between relatively early postoperative endothelial cell loss and unfolding time, with longer unfolding time associated with greater endothelial cell loss.^3^ Another study found no correlation between corneal donor characteristics and the degree of difficulty of unfolding with graft lamella older than 49 years. The same study indicated that there was a significant association between more difficult graft unfolding and rates of graft detachments and endothelial cell loss.^14^ It was reported that graft orientation in DMEK surgery can be visualized and assessed with live intraoperative optical coherence tomography. In addition, faster graft positioning with less graft manipulation was reported in the presence of severe corneal edema.^15^ Different ways to facilitate graft unfolding are still being sought.

When the DMEK graft is separated from the corneal stroma, it forms a roll with the DM inside and the endothelium facing outward. This formation is associated with type IV collagen in the DM and the behaviors of endothelial cells after leaving the cornea. More studies which demonstrate how graft properties affect graft unfolding time are needed. We were unable to find any animal model or other research in the literature showing that temperature change can affect DM roll tightness.

As the prognosis in the postoperative period depends on the remaining number of endothelium cells, DMEK surgery and DM unfolding times are important. The DM roll should be opened in the anterior chamber with minimum endothelial loss and attached to the recipient stroma. Recently, several maneuvers for atraumatic unrolling of DMEK grafts have been described.^16,17,18^ An *in vitro* study showed that delivering DMEK tissue trifolded with the endothelium inward reduced surgical trauma to donor cells and facilitated spontaneous unfolding.^17^ However, despite the maneuvers developed, there is no standardized method of graft unfolding which affects postoperative success, and DM unfolding time can be prolonged in some cases. Moreover, the factors affecting DM unfolding time have not been determined. Existing methods used for DM unfolding result in direct or indirect mechanical trauma to the graft, which can cause endothelium injury. Therefore, less traumatic methods need to be developed.

In some cases where we had difficulty in DM unfolding, in time we observed spontaneous unfolding. Supposing that this could be associated with heating under the microscope light, we put DM rolls obtained from this study into different BSS temperatures and observed whether any would open spontaneously. We refrained from any other maneuvers that would affect opening and investigated only the effect of the temperature. However, no changes were observed in DM rolls at 8 °C, 16 °C, 23 °C (BSS temperature we use routinely) or 36 °C BSS temperatures within the 10-minute period.

The DM rolls demonstrated neither tightening nor opening in our study. We had anticipated that 36 °C, which is normal physiological body temperature, would promote DM roll unfolding. However, we did not observe such an effect. Although we did not demonstrate the effect of BSS temperature on the DM rolls at the molecular level, it was observed that this effect did not change unfolding time of the graft. In a study using porcine corneal endothelial cells, it was found that the eye irrigation solution stored in the refrigerator better protected the corneal endothelial cells from heat damage than BSS stored in air-conditioned room.^19^ Consequently, using BSS at a lower temperature may be advantageous and rational for DM graft since the higher BSS temperature offers no benefit in terms of DM graft unfolding time.

### Study Limitations

This study has some limitations. First, we used biomicroscopic examination to detect endothelial pathology and we could not count endothelial cells. If we had a chance to perform specular microscopy, endothelial examination would be more valuable than biomicroscopic examination. Second, delivery of the tissue could not be simulated using an anterior chamber.

## Conclusion

In conclusion, different BSS temperatures were found to have no effect on DM unfolding time in this study. Other methods which are less traumatic and facilitate DM unfolding should be investigated in order to reduce endothelial cell loss.

## Figures and Tables

**Table 1:**
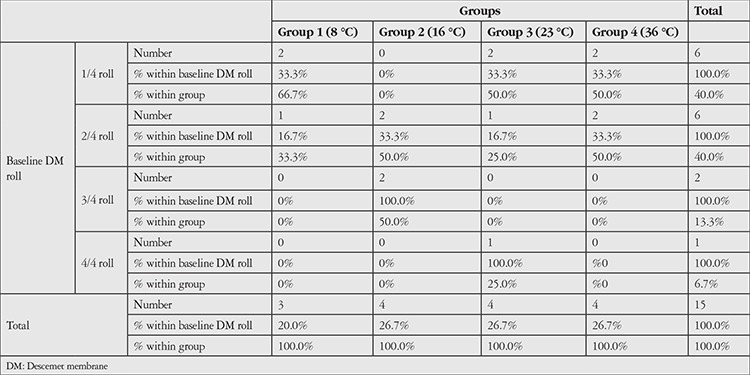
Distributions of the Descemet membrane roll formation between the groups at the beginning
